# 307. Predictors of Respiratory Bacterial Co-Infection in Hospitalized COVID-19 Patients

**DOI:** 10.1093/ofid/ofab466.509

**Published:** 2021-12-04

**Authors:** Erica E Reed, Austin Bolker, Kelci E Coe, Jessica M Smith, Kurt Stevenson, Shu-Hua Wang

**Affiliations:** 1 The Ohio State University Wexner Medical Center, Columbus, OH; 2 Ohio State University Wexner Medical Center, Columbus, OH; 3 The Ohio State University College of Medicine and College of Public Health, Columbus, Ohio

## Abstract

**Background:**

COVID-19 pneumonia can be indistinguishable from other infectious respiratory etiologies, so providers are challenged with deciding whether empiric antibiotics should be prescribed to hospitalized patients with SARS-CoV-2. This study aimed to evaluate predictors of respiratory bacterial co-infections (RBCI) in hospitalized patients with COVID-19.

**Methods:**

Retrospective study evaluating COVID-19 inpatients from Feb 1, 2020 to Sept 30, 2020 at a tertiary academic medical center. Patients with RBCI were matched with three COVID-19 inpatients lacking RBCI admitted within 7 days of each other. The primary objectives of this study were to determine the prevalence of and identify variables associated with RBCI in COVID-19 inpatients. Secondary outcomes included length of stay and mortality. Data collected included demographics; inflammatory markers; bacterial culture/antigen results; antibiotic exposure; and COVID-19 severity. Wilcoxon rank sum, Chi Square tests, or Fisher’s exact tests were utilized as appropriate. A multivariable logistic regression (MLR) model was conducted to identify covariates associated with RBCI.

**Results:**

Seven hundred thirty-five patients were hospitalized with COVID-19 during the study period. Of these, 82 (11.2%) had RBCI. Fifty-seven of these patients met inclusion criteria and were matched to three patients lacking RBCI (N = 228 patients). Patients with RBCI were more likely to receive antibiotics [57 (100%) vs. 130 (76%), p < 0.0001] and for a longer cumulative duration [19 (13-33) vs. 8 (4-13) days, p < 0.0001] compared to patients lacking RBCI. The MLR model revealed risk factors of RBCI to be admission from SNF/LTAC/NH (AOR 6.8, 95% CI 2.6-18.2), severe COVID-19 (AOR 3.03, 95% CI 0.78-11.9), and leukocytosis (AOR 3.03, 95% CI 0.99-1.16).

**Conclusion:**

Although RBCI is rare in COVID-19 inpatients, antibiotic use is common. COVID-19 inpatients may be more likely to have RBCI if they are admitted from a SNF/LTAC/NH, have severe COVID-19, or present with leukocytosis. Early and prompt recognition of RBCI predictors in COVID-19 inpatients may facilitate timely antimicrobial therapy while improving antimicrobial stewardship among patients at low risk for co-infection.

**Disclosures:**

**All Authors**: No reported disclosures

